# What Drives Embryo Development? Chromosomal Normality or Mitochondria?

**DOI:** 10.1155/2017/4397434

**Published:** 2017-08-08

**Authors:** A. Bayram, I. Elkhatib, A. Arnanz, A. Linan, F. Ruiz, B. Lawrenz, H. M. Fatemi

**Affiliations:** ^1^IVI Middle East, IVF-Laboratory, Royal Marina Village, Villa B22-23, Abu Dhabi, UAE; ^2^IVI Middle East, IVF-Clinic, Royal Marina Village, Villa B22-23, Abu Dhabi, UAE; ^3^Women's University Hospital Tuebingen, Calwer Str. 7, Tuebingen, Germany

## Abstract

**Objective:**

To report the arrest of euploid embryos with high mtDNA content.

**Design:**

A report of 2 cases.

**Setting:**

Private fertility clinic.

**Patients:**

2 patients, 45 and 40 years old undergoing IVF treatment.

**Interventions:**

Mature oocytes were collected and vitrified from two ovarian stimulations. Postthaw, survived mature oocytes underwent fertilization by intracytoplasmic sperm injection (ICSI). Preimplantation genetic screening (PGS) and mitochondrial DNA (mtDNA) copy number were done using next generation sequencing (NGS). The only normal embryo among the all-biopsied embryos had the highest “Mitoscore” value and was the only arrested embryo in both cases. Therefore, the embryo transfer was cancelled.

**Main Outcome Measures:**

Postthaw survival and fertilization rate, embryo euploidy, mtDNA copy number, and embryo development.

**Results:**

In both patients, after PGS only 1 embryo was euploid. Both embryos had the highest mtDNA copy number from all tested embryos and both embryos were arrested on further development.

**Conclusions:**

These cases clearly demonstrate the lack of correlation between mtDNA value (Mitoscore) and chromosomal status of embryo.

## 1. Introduction

Age-related decline in female fertility is primarily due to the decrease in oocyte quantity and quality and increase in aneuploidy rate rather than changes in endometrial receptivity, as indicated by data obtained from oocyte donation cycles [1, 2].

Mammalian oocytes are long-lived cells in the human body. They initiate meiosis already in the embryonic ovary and arrest meiotically for long periods [3]. Ovarian aging is characterized by quantitative and qualitative alteration of the ovarian oocyte reserve [4].

Mitochondria play a central role in follicular atresia and could be the main target of the ooplasmic factors determining oocyte quality adversely affected by aging. Indeed, the oocyte is the richest cell of the body in mitochondria and depends largely on these organelles to acquire competence for fertilization and early embryonic development [4]. The assessment of mtDNA quantity with PGS has been proposed to be a novel way of identifying embryos with the highest ability to lead to healthy pregnancies and live births. “Mitoscore” is a value that represents the normalized mtDNA content in embryos and indicates the total mtDNA content in the sample [5, 6].

## 2. Material and Methods

We describe herein 2 cases of patients, who were treated at our center for primary infertility, where a lack of correlation between mtDNA content and chromosomal information of the embryos could be observed.

### 2.1. Case 1

45-year-old patient presented with a history of 8 years of primary infertility. She had undergone elsewhere one ICSI-treatment without an embryo transfer due to poor embryo development and high fragmentation.

In order to improve the patient's chance for a pregnancy and due to the patient's age, repeat ovarian stimulation for oocyte accumulation [7] with subsequent ICSI-procedure, testing of the embryo with PGS (Preimplantation Genetic Screening), and evaluation of Mitoscore were recommended [8]. Recommendations were given, taking into consideration the country's laws and regulation, which forbids oocytes donation and embryo freezing.

After two stimulation cycles, using long GnRH (Gonadotropin-Releasing Hormone) agonist protocol, a total of 24 metaphase II oocytes were vitrified. Embryo transfer was planned in a warmth oocyte embryo transfer cycle (WOET), using a hormonal replacement cycle for endometrial preparation.

When the lining appeared with an adequate thickness and pattern, all 24 metaphase II vitrified oocytes were thawed, all survived, and ICSI was performed. 21 oocytes were successfully fertilized (2PN-pronucleus) and one 3PN. The embryos showed high fragmentation on day 2 and slow division on day 3. Day 3 biopsy was done for 11 embryos that had developed to more than 4 cells on day 3 [9]. On day 5, 1 embryo developed to an early blastocyst, 3 embryos developed to compacting morula, 1 embryo developed to 9-cell stage, and 6 embryos were arrested. PGS showed 10 aneuploid embryos and 1 euploid embryo. The embryo with the highest Mitoscore value was the only euploid one; however it was arrested (Table 1). No embryo transfer was done. The 10 aneuploid embryos were annotated on day 5 as follows: 1 was early blastocyst, 3 were compacting morulae, and 6 were cells' stage.

### 2.2. Case 2

The second case is 40-year-old patient with primary infertility due to obstructive azoospermia since 2 years with 8 failed attempts. The patient underwent 2 stimulation cycles in a GnRH-antagonist protocol; however there was no euploid embryo after PGS, so no embryo transfer was done. 2 additional stimulation treatments in a GnRH-antagonist protocol were performed for oocyte accumulation and with both stimulations, a total of 18 COCs were collected and 17 were metaphase II and vitrified.

Embryo transfer was planned in a warmth oocyte embryo transfer cycle (WOET), using a hormonal replacement cycle for endometrial preparation. When the lining appeared with an adequate thickness and pattern, the oocytes were thawed. On the day of oocyte thawing, fresh fine needle aspiration (FNA) was performed. 15 oocytes survived after thawing and all were injected with motile sperm. 10 were normally fertilized. PGS using NGS was done for 9 embryos. 8 were abnormal and one was normal. The only normal embryo had the highest Mitoscore value and was the only one arrested (Table 2). Embryo transfer was not done due to poor embryo development. The 8 aneuploid embryos were annotated on day 5 as follows: 4 embryos reached the early blastocyst stage, 2 were compacting morulae, and 2 were 7 and 10 cells.

## 3. Discussion

Earlier studies examining human mitochondria and mtDNA in relation to ovarian aging have focused on the analysis of oocytes rather than embryos. Most of them reported that mtDNA copy number either remains unchanged or decreases in older women [10]. A retrospective study found that high mtDNA copy number in euploid embryos is indicative of lower embryo viability in terms of implantation potential [5].

In our first case, the only euploid embryo in the cohort had a Mitoscore value of 505.26 which is a very high number. Similarly in the second one, the only normal embryo's Mitoscore value was 181.10 as the highest one in the all-biopsied embryos.

Data from single euploid embryo transfers with mtDNA analysis support the hypothesis that mtDNA copy number in the embryo is not a direct indicator of energetic capability, rather it is an index of energetic stress and thus it can potentially be used to predict the implantation capacity [5]. Elevated mtDNA levels are most likely a consequence of a compensating mechanism to normalize adenosine triphosphate (ATP) generation resulting from organelles with reduced function. Mitochondria in the oocytes of older hamsters and mice have been shown to generate higher levels of reactive oxygen species (ROS) and produce less ATP, which indicates less support towards dynamic processes, such as preimplantation development [11]. In humans, if this scenario occurs, an increase in mitochondrial number may be needed in the embryos of older women, in order to maintain the required ATP levels.

Despite the fact that the embryos in the here presented cases were euploid, they showed a poor development, represented as slow division and high fragmentation. Many of them ended with developmental arrest. Biopsies were done on day 3 and results were obtained on day 5 morning. Although one could argue on the limitations of day 3 biopsy, it has been shown that day 3 embryo biopsies can be representative of the whole embryo; hence, it can be used for clinical analysis in PGS [12].

Developmental arrest is the early embryonic cessation in utero before normal completion and ROS is involved in this process [13]. About 90% of cellular ROS is produced by the mitochondria [14, 15] and high levels of ROS are associated with mtDNA damage. Obviously, mtDNA is particularly vulnerable due to its proximity to the source of oxidants. Initially, ROS induces impairment of mitochondria and therefore it leads subsequently to increased oxidant production, which in turn results in further mitochondrial damage. Old mitochondria appear morphologically and functionally altered and produce more oxidants and less ATP [16].

Possibly, embryos developed from older oocytes are going through a certain degree of stress and therefore require more energy. Elevated values of Mitoscore might be associated with increased metabolic requirements of the embryo. It may be that mitochondrial metabolism is indeed effective on the accuracy of chromosome segregation, since ATP is required for correct oocyte spindle assembly and chromosome alignment.

In our cases, the euploid embryos showed elevated Mitoscore values, whereas Fragouli's study showed an increased tendency of mtDNA in aneuploidy embryos. These contrary findings demonstrate the existing lack of information about direct relationship between elevated mtDNA content and aneuploidy.

## 4. Conclusion

These cases demonstrate the possible relationship between mtDNA content, female age, and embryo development, despite a lack of correlation between Mitoscore and chromosomal information of the embryo. Since a significant percentage of morphologically and chromosomally normal embryos fail to implant, future studies should evaluate whether the abnormal mtDNA levels in nonimplanting euploid embryos may be one of the causes of the implantation failure.

Studies have shown that the lower the Mitoscore values, the higher the implantation rate [5], and euploid embryos have higher implantation rate [17]; that is, that euploid embryos would have lower Mitoscore values.

The current cases clearly indicate that there seem to be no correlation between the Mitoscore and the chromosomal status of the embryo, as evaluated by a day 3 biopsy.

Future randomized controlled trials should evaluate the possible correlation between embryo chromosomal status and the mtDNA content and access whether the mtDNA does have any correlation with the chromosomal status of the embryo.

## Figures and Tables

**Table 1:** Case 1.

Embryo number	Mitoscore	PGS result
1	62.43	Abnormal
6	86.86	Abnormal
7	49.4	Abnormal
9	61.73	Abnormal
10	505.26	Normal
11	51.26	Abnormal
15	48.23	Abnormal
19	76.16	Abnormal
21	43.11	Abnormal
22	42.18	Abnormal
23	66.62	Abnormal

**Table 2:** Case 2.

Embryo number	Mitoscore	PGS result
1	71.74	Abnormal
2	67.08	Abnormal
6	99.43	Abnormal
8	100.36	Abnormal
9	89.89	Abnormal
10	181.1	Normal
12	83.6	Abnormal
13	95.47	Abnormal
15	59.87	Abnormal
